# Efficacy Oral Glutamine to Prevent Oral Mucositis and Reduce Hospital Costs During Chemotherapy in Children with Acute Lymphoblastic Leukemia

**DOI:** 10.31557/APJCP.2020.21.7.2117

**Published:** 2020-07

**Authors:** Nur Aisiyah Widjaja, Ardha Pratama, Rendi Aji Prihaningtyas, Roedi Irawan, IDG Ugrasena

**Affiliations:** *Department of Child Health, Faculty of Medicine, Universitas Airlangga, Dr. Soetomo General Academic Hospital, Surabaya, Indonesia. *

**Keywords:** Methotrexate, glutamine, mucositis, chemotherapy, acute lymphoblastic leukemia

## Abstract

**Objective:**

To investigate the use of glutamine administered orally during Methotrexate chemotherapy to prevent oral mucositis and reduce hospital costs in children with acute lymphoblastic leukemia (ALL).

**Methods:**

Twenty-four children received oral glutamine (400 mg/kg body weight per day) and twenty four received placebo on days of chemotherapy administration and for at least 14 additional days. Oral mucositis was graded daily at each day of treatment till completion of therapy. The study groups were compared for the oral mucositis development using the WHO scale.

**Results:**

Oral mucositis occurred in 4.2 % of the glutamine group and 62.5% in the placebo group. The use of glutamine was directly associated with prevention of oral mucositis than placebo (OR 0,026; 95% CI: 0,003-0,228). The duration of length hospital stay was lower in the glutamine group than in the placebo group ((8 vs 12 days); p = 0,005). Hospital cost per day for glutamine group was 40 USD per day while placebo group was 48 USD per day.

**Conclusions:**

There was significant difference in the prevention of oral mucositis by oral glutamine vs placebo. The hospital cost for glutamine supplementation was lower than control group.

## Introduction

Leukemia is the most common malignancy in children with a prevalence of 30-40%. The incidence of leukemia reaches 2.76 / 100,000 in children aged 1-4 years with the most cases are acute lymphoblastic leukemia (ALL) (Permono et al., 2012). The highest overall incidence of oral mucositis in a study can reach 80-100% and 25 - 45% of them experience severe oral mucositis (stage 3 or 4). High-dose methotrexate in the ALL chemotherapy protocol has been shown to significantly increase the risk of oral mucositis which is a reactive process of interaction between environmental damage to the oral cavity, bone marrow suppression, infection and as a side effect of chemotherapy (Sukhotnik et al., 2014).

Glutamine acts to accelerate the division of leukocytes along with macrophages in the immune system. Glutamine stimulates the synthesis of hexosamine as a mucin-forming material that lines the mucosa and as a barrier in preventing bacterial translocation. The use of glutamine in preventing oral mucositis in chidren with ALL after high-dose methotrexate chemotherapy is widely introduced but there is no agreement on the terms of prevention and treatment of oral mucositis (Naidu et al., 2004; Silverman 2007; Chang et al., 2017). Glutamine supplementation can reduce the incidence and severity of oral mucositis during treatment by repairing cell damage and cell recovery (Chang et al., 2017). However, other studies have not found definitive results, and show that more research with higher methodological consistency and validity is needed to find more solid evidence.

Mucositis will affect nutrient intake, energy requirements, speech difficulties, and decrease or increase in saliva, ulcerations that cause systemic infections, delay chemotherapy, extend treatment days (treatment costs), affect quality of life and mortality (Sonis, 2004; Silverman, 2007; Vadhan-Raj, 2010). Glutamine appears to be appropriate and safe for preventing oral mucositis and can be considered in patients receiving high doses of methotrexate (Chang et al., 2017). However, to date there has been no definitive guidance in the management of oral mucositis after the administration of high-dose methotrexate in pediatric ALL chemotherapy. Therefore, research on the use of glutamine in the prevention of oral mucositis is very important to be done in children undergoing ALL chemotherapy with the administration of high doses of methotrexate.

The aim of this study was to evaluate the incidence of oral mucositis, duration of treatment and cost of care in children with ALL who received high-dose methotrexate chemotherapy in the consolidation phase with glutamine administration at Dr. Soetomo General Academic Hospital, Surabaya.

## Materials and Methods

The research design used was an experimental study with a randomized pre-post test controlled group trial. The researcher and the research subject (S) were blind fot the treatment (type of drug) given. Subjects grouped into two random groups (R) namely the control group (C) who received a placebo and the treatment group (T) who received oral glutamine. Researchers and research subjects did not know which preparations to be given were glutamine or placebo (double blind) (Figure 1).

Subjects were children with ALL aged 1 to 18 years who underwent consolidation phase chemotherapy and received high-dose methotrexate according to the 2013 Indonesian ALL Chemotherapy Protocol and did not experience allergies. All subjects will receive ALL chemotherapy using the 2013 Indonesian ALL Chemotherapy Protocol for the Consolidation Phase of high-dose methotrexate. 

The study subjects were 48 children divided into two treatment groups: the control group, given a placebo (24 subjects) and the treatment group (24 subjects) were given glutamine at a dose of 400 mg/kg/day orally. Glutamine and placebo were started 24 hours before high-dose methotrexate chemotherapy was given according to protocol. Glutamine and placebo were given for 14 days. The incidence of mucositis and the degree of mucositis were evaluated using the WHO’s Oral Toxicity Scale on days 3, 4, 5, 6, 7 and 14 after administration of high-dose methotrexate (O1 and O2) by dental and oral specialists as shown in Figure 1.

## Results

Characteristics of the sample in the homogeneous study and control group in the age group, sex, nutritional status and diagnosis (p> 0.05) as shown in Table 1. The ratio of male and female sex ratio in the glutamine group was 31/17. There were more ALLs in boys than girls (Rubenstein et al., 2004).

ALL subjects who participated in the glutamine group had the nutritional status, including 37.5% underweight, 4.2% overweight and 8.3% obesity. Whereas in the placebo group there were children with 29.2% underweight, 12.5% overweight and 16.7% obesity (Table 1).

In the treatment group, 23 subjects suffered from oral mucositis, whereas in the placebo group found 15 subjects suffered from oral mucositis. Comparative analysis of the incidence of oral mucositis showed a statistically significant difference (p <0.05). Odds ratio of glutamine administration to the incidence of mucositis in children with ALL with high-dose methotrexate therapy was 0.026. This study showed the results of statistical tests using the Mann-Whitney Test for the incidence of oral mucositis were significantly lower in the glutamine group than in the control group (4.2% vs 62.5%; p value <0.05) (Table 2).

In the treatment group, the stage of oral mucositis was lower than in the placebo group (Table 3). There were no subjects in the glutamine group suffered from oral mucositis (stages 3 and 4). In one incident oral mucositis obtained in the glutamine group occurred on the 3rd day which was the first day of initial observation with a severity of stage 2 where the patient was experiencing pain, mild ulcers and subjects were still able to swallow solid food, then on the fourth and fifth day observations there was improvement in oral mucositis to stage 1 and oral mucositis was not obtained on the 6^th ^day of observation until the 14^th^ day until continued 2 weeks after supplementation. No recurrence of oral mucositis was reported from the patient’s parents. The highest peak in this study was on the 4th day. This study suggested that glutamine can reduce the incidence and severity of oral mucositis in ALL children receiving high-dose methotrexate chemotherapy (Table 4).

Glutamine administration affected the duration of treatment, in which the glutamine group had shorter treatments (7.67 days + 0.59 SD) than the placebo group (12 days + 2.57 SD). This also affected the amount of costs incurred, where the glutamine group had a total cost that was almost 2 times lower (Rp. 4,709,828 + SD 9,049) than the placebo group (Rp. 9,336,405 + SD 2,517), as in Table 5 .

**Table 1:** Subject Characteristics

	Glutamine	Plasebo	p value
	n = 24	n = 24	
Age, Mean (SD) year	6.29 (4.42)	5.9 (2.9)	0.651
Age group			0.836
1 – 5 years old	13 (54.2%)	10 (41.7%)	
5 – 11 years old	6 (25%)	13 (54.2%)	
11 – 18 years old	5 (20.8%)	1 (4.2%)	
Sex			1.000
Male	16 (66.7%)	15 (62.5%)	
Female	8 (33.3%)	9 (37.5%)	
Nutritional Status			0.233
Underweight	9 (37.5%)	7 (29.2%)	
Normal	12 (50%)	10 (41.7%)	
Overweight	1 (4.2%)	3 (12.5%)	
Obesity	2 (8.3%)	4 (16.7%)	
Diagnosis			0.238
ALL-SR	7 (29.8%)	12 (50%)	
ALL-HR	17 (70.2%)	12 (50%)	

ALL-SR, Acute Lymphoblastic Leukemia-Standard Risk; ALL-HR, Acute Lymphoblastic Leukemia-High Risk

**Table 2:** Occurrence of Oral Mucositis during Administration of High-Dose Methotrexate

	Glutamin	Plasebo	p value
	n = 24	n = 24	
Mucositis	1 (4.2%)	15 (62.5%)	0.001*
No mucositis	23 (95.8%)	9 (37.5%)	

*p < 0.05

**Table 3:** Oral Mucositis Stage based on WHO’s Oral Toxicity Scale during Administration of High-Dose Methotrexate

	Glutamine	Plasebo
	n = 24	n = 24
Oral Mucositis		
Stage 1	-	1 (4.2%)
Stage 2	1 (4.2%)	6 (25%)
Stage 3	-	7 (29.2%)
Stage 4	-	1 (4.2%)

**Table 4:** The Stage of Clinical Manifestation Using WHO's Oral Toxicity Scale during High-Dose during Methotrexate Administration for 14 Days Observation

	Day-0 (n = 24)		Day-3 (n = 24)		Day-4 (n = 24)		Day-5 (n = 24)		Day-6 (n = 24)		Day-7 (n = 24)		Day-14 (n = 24)	
	G	P	G	P	G	P	G	P	G	P	G	P	G	P
Stage 1	-	-	-	3	1	2	1	2	-	4	-	4	-	1
Stage 2	-	-	1	7	-	10	-	9	-	7	-	6	-	-
Stage 3	-	-	-	4	-	3	-	4	-	3	-	3	-	1
Stage 4	-	-	-	-	-	-	-	-	-	1	-	1	-	1

*G, Glutamine group; P, Plasebo

**Table 5:** Duration of Treatment and Costs Incurred by Research Subjects

Treatment	Lenght of Hospital Stay (day)	Costs per day (Rp.)	Total Costs (Rp.)
Glutamin group (n=24)	7.67 + SD 0.59	586.547,- + SD 5.376 (40 USD)	4.709.828,- + SD 9.049 (314 USD)
Placebo (n=24)	12 + SD 2.57	671.348,- + SD 5.517 (48 USD)	9.336.405 + SD 2.517 (622 USD)

## Discussion

Nutritional status is important in terms of its effect on long-term prognosis and also as a predisposition to the incidence of infection. Leukemic children with poor and poor nutrition are more prone to experience toxicity that affects the occurrence of mucositis (Niscola et al., 2007). Oral mucositis can be very painful and significantly affect nutrition intake, oral health and quality of life (Duncan et al., 2005; Lalla et al., 2008) and experience a weight loss of> 5% (Lalla and Peterson 2005). Mucositis also affects energy requirements, speech difficulties, and decreased or increased saliva, ulcerations that cause systemic infections and delayed chemotherapy. This condition will affect the nutritional condition of the patient (Sonis 2004; Silverman 2007; Vadhan-Raj 2010).

In this study 62.5% of subjects in the placebo group experienced mucositis after the administration of high-dose methotrexate, while the treatment group receiving glutamine experienced 4.2% mucositis. Oral mucositis is the most common complaint arising from administration of high doses of methotrexate, but until now there has been no effective therapy in the prevention and treatment of oral mucositis (Petersen 2005). Previous studies showed glutamine supplementation can reduce the incidence and severity of mucositis during chemotherapy by repairing cell damage and cell recovery (Chang et al., 2017).

Biological formation of mucositis is found in five phases: (a) the initiation phase in which chemotherapy acts as a free radical that can damage DNA; (b) The message generation phase in which the activation of transcription factors (NF-kβ) regulates the amount of pro-inflammatory cytokine / interleukin 1 beta (IL-1β) and tumor necrosis factor-alpha (TNF-α). Cytokines IL-1β play a role for inflammation and dilation of blood vessels so that it is likely to increase the concentration of chemotherapy in the area, and TNF-α causes tissue damage; (c) The signaling and amplification phase in which TNF-α activates NF-kβ, mitogen activated protein kinase (MAPK), and sphingomyelinase pathways that magnify cell and tissue damage resulting in erythema and epithelial atrophy 4-5 days after the initial stage of chemotherapy. Minor trauma from daily activities such as swallowing and chewing can cause ulceration; (d) Ulceration / bacteriological phase if neutropenia is suspected to occur bacterial colonization of the ulcer so that in the mucosal tissue there are many endotoxins and the subsequent release of IL-1 and TNF-alpha; (e) The healing phase in which re-epithelialization occurs in the ulcer (Sonis, 2004).

In these conditions glutamine will accelerate the division of leukocytes and macrophages in the body’s immune system, stimulating the synthesis of hexosamine as a mucin-forming material that lines the mucosa as well as a barrier in preventing bacterial translocation. Glutamine is an immune system fuel that stimulates IgA which produces plasma cells through IL-4 and IL-10 which produce IgA. SIgA is IgA which is related to the secretory component. Mucosal immune system will increase preventing bacterial translocation and sepsis (Neu, 2001).

Methotrexate in experimental animals histologically showed significant damage to the shape of the crypt and improvement signs of the crypt, severe damage to the epithelial villi with atrophy, degeneration and shortening of the size of the villus, and infiltration of polymorphonuclear leukocytes in the lamina propria (Sukhotnik et al., 2014). Giving glutamine can prevent mucositis in children with ALL who receive high-dose methotrexate therapy. The exact mechanism of glutamine’s role in preventing mucositis remains unclear. Glutamine is thought to be the main oxidative fuel of the gastrointestinal epithelium and helps maintain the integrity of the intestinal structure under normal conditions and stress conditions, so glutamine is very useful in preventing mucositis in patients who are at risk of developing mucositis (Klimberg et al., 1990). Significantly glutamine is provided by skeletal muscle during hyper-catabolic conditions such as cancer. This causes glutamine to be depleted excessively (Luo et al., 2014). Under depleted conditions, glutamine synthesis cannot replace the amount of glutamine lost (Sayles et al., 2016).

Until now there has been no research that found dangerous side effects from the use of glutamine supplementation even in large doses, because glutamine is an amino acid that is naturally present in the human body and is well regulated by the body (Sonis, 2004). The duration of mucositis can increase the severity of mucositis, duration of pain, opioid use, dysphagia, total use of parenteral nutrition, incidence and/or duration of fever and infection, and duration of antibiotic use (McCann et al., 2009). The degree of mucositis is significantly related to the duration of parenteral drug administration and TPN, the incidence of infection, the length of stay and the total cost of hospitalization (Vera-Llonch et al., 2007).

Giving Glutamine can reduce the length of patient care in the hospital, because glutamine is able to prevent oral mucositis and reduce the duration and severity of mucositis. the incidence of mucositis prolongs the length of treatment, and oral glutamine administration provides benefits for overcoming and shortening treatment days (Chang et al., 2017). In medical intervention in reducing the incidence and severity of mucositis, the duration of treatment is significantly shorter (Suzuki et al., 2014). The duration of treatment is influenced by the severity of the disease. Mucositis extended the length of treatment by 2.3 days (McCann et al., 2009).

In this study, the cost of treating patients who received glutamine decreased significantly, because patients who received chemotherapy and had mucositis needed supportive care such as total parenteral nutrition, fluid replacement, and prophylaxis against infection. Oral mucositis is a significant toxicity and limits the dose of cancer therapy, with clinical and economic implications (Lalla et al., 2008). This is in line with previous research which showed that mucositis affects the cost of hospitalization for patients, including the cost of a doctor’s visit, the cost of a nutritionist and the cost of pain reduction therapy (Murphy et al., 2009).

A one-point increase in mucositis scores is associated with an increase in days of fever, a 2.1-fold increase in risk of infection, an additional 2.7 days of TPN, an additional 2.6 days of injection of narcotic therapy, an additional 2.6 days of hospitalization and a 3.9-fold increase in the risk of death by 100 days, collectively contributing more than $ 25,000 in additional hospital costs (Lalla et al., 2008).

In conclusion, this study is the first RCT in Indonesia that evaluates the administration of oral glutamine at a dose of 400mg/kg BW/day against the prevention of oral mucositis in children with acute lymphoblastic leukemia receiving high-dose methotrexate chemotherapy in the consolidation phase. Oral glutamine dose of 400 mg/kg/day can prevent oral mucositis, shorten the length of treatment and reduce the cost of care for ALL child patients due to the administration of high-dose methotrexate chemotherapy in the consolidation phase
